# Determinants of Pregnancy-Induced Hypertension among Mothers Attending Public Hospitals in Wolaita Zone, South Ethiopia: Findings from Unmatched Case-Control Study

**DOI:** 10.1155/2021/6947499

**Published:** 2021-10-28

**Authors:** Yitagesu Belayhun, Yibeltal Kassa, Niguse Mekonnen, Wakgari Binu, Mahilet Tenga, Bereket Duko

**Affiliations:** ^1^Health Development Planning and Economic Administration, South Nations Nationalities and People Regional State Health Bureau, Hawassa, Ethiopia; ^2^School of Public Health, College of Health Sciences and Medicine, Wolaita Sodo University, Wolaita Sodo, Ethiopia; ^3^Medical Services Directorate, South Nations Nationalities and People Regional State Health Bureau, Hawassa, Ethiopia; ^4^Faculty of Heath Sciences, College of Medicine and Health Sciences, Hawassa University, Awasa, Ethiopia; ^5^Curtin School of Population Health, Faculty of Health Sciences, Curtin University, Perth, Australia

## Abstract

**Background:**

It has been estimated that approximately 14% of maternal death has resulted due to pregnancy-induced hypertension. Evidence also suggests that pregnancy-induced hypertension may result in adverse maternal and child outcomes. The aim of this study was to assess the determinants of pregnancy-induced hypertension among mothers attending antenatal and delivery services at public health hospitals in Wolaita zone, southern Ethiopia.

**Methods:**

An institutionally based unmatched case-control study was conducted at three public hospitals. A total of 283 study participants were recruited for this study. Cases were selected consecutively as they were being diagnosed for pregnancy-induced hypertension, and two controls were selected for each case. Data were collected via the face-to-face interview technique using a pretested questionnaire. Unconditional logistic regression analysis was used to identify the independent predictor variables and produced odds ratio (OR) as a measure of association.

**Results:**

The mean ± (SD) ages of cases and controls were 26.1 ± 5.4 and 26.1 ± 4.5 years, respectively. Being rural residents (AOR: 2.25, 95% CI: 1.09–4.65), illiterate (AOR: 3.12, 95% CI: 1.20–8.08), having the history of pregnancy-induced hypertension (AOR: 6.62, 95% CI: 2.48–17.71), history of kidney disease (AOR: 3.14, 95% CI: 1.05–9.38), and family history of hypertension (AOR: 5.59, 95% CI: 2.73–11.45) were determinants that increased the odds of suffering from hypertensive disorders of pregnancy. More importantly, eating vegetables and fruit reduces the odds of suffering from pregnancy-induced hypertension by 77% (AOR: 0.23, 95% CI: 0.06–0.79).

**Conclusion:**

Being rural residents, illiterate, having a history of pregnancy-induced hypertension, and history of kidney disease, as well as the family history of hypertension were identified determinates of hypertensive disorders of pregnancy in the study area. Furthermore, fruit and vegetable intakes were identified as protective factors for pregnancy-induced hypertension. Therefore, early diagnosis and intervention of this disorder are warranted to reduce adverse outcomes.

## 1. Background

Pregnancy-induced hypertension is the most common medical disorder of pregnancy that complicates 6–10% of pregnancies all around the world. It is the second direct cause of maternal mortality globally [1]. It is defined as either a systolic BP of 140 mm Hg or greater, a diastolic BP of 90 mm Hg or greater, or both [2]. Though there is no clear agreement on the definition and classification of pregnancy-induced hypertension due to limited knowledge of the etiology of pregnancy-induced hypertension and the continuous nature of the signs and symptoms used for the diagnosis, the American College of Obstetricians and Gynecologists (ACOG) Task Force on Hypertension in Pregnancy classifies pregnancy-induced hypertension into four categories that includes preeclampsia-eclampsia, chronic hypertension, chronic hypertension with superimposed preeclampsia, and gestational hypertension [3].

Globally, nearly 350,000 mothers die each year due to pregnancy-related causes, and 14% of maternal deaths is due to pregnancy-induced hypertension [1]. Regional estimates varied substantially, and the large proportion of deaths occurred in sub-Saharan Africa and southeast Asia [1, 4, 5]. In Ethiopia, the pooled prevalence of pregnancy-induced hypertension is 6.25% with a higher prevalence of pregnancy-induced hypertension in Southern Nations, Nationalities, and Peoples' of Ethiopia which is 10.3% [6].

Pregnancy-induced hypertension especially preeclampsia and eclampsia are significant contributors to the global burden of maternal and perinatal mortality [4, 7]. It remains one of the top causes of maternal mortality and morbidity in high, middle, and low-income countries [7]. In Ethiopia, it is the third main direct cause of death next to hemorrhage and obstructed labor/ruptured uterus, specifically preeclampsia contributes to the 78% of maternal deaths [7, 8]. Pregnancy-induced hypertension also increases neonatal adverse outcomes such as preterm birth, intrauterine growth restriction, low birth weight, high neonatal admissions, and intrauterine and perinatal death [6, 9, 10]. The risk factors of pregnancy-induced hypertension include demographic, familial factors, medical/obstetric history, current pregnancy history, and paternal factors. These factors are used to identify women at increased risk of hypertensive disorders of pregnancy that warrants enhanced surveillance and therapy [2].

The government of Ethiopia has implemented different strategies to improve maternal health through increasing demand for maternal health services and easier access to basic and essential obstetric services, expansion of health facilities, increasing availability of supplies, and deployment of skilled health professionals [11]. Despite such efforts and strategies, pregnancy-induced hypertension is still the significant contributor of maternal and neonatal mortality and morbidity globally including Ethiopia [3, 12]. Few prevalence studies were conducted on the causes of pregnancy-induced hypertension in southern Ethiopia, and as per the researcher's best literature search and review, there was no study conducted in Wolaita zone, southern Ethiopia. Therefore, the aim of this study was to identify the main determinants of pregnancy-induced hypertension among mothers attending antenatal and delivery care services at selected public hospitals of Wolaita zone, southern Ethiopia.

## 2. Methods

### 2.1. Study Setting and Population

An institutionally based unmatched case-control study was conducted at three public hospitals in Wolaita zone, southern Ethiopia. Wolaita zone is located at 328 km south of the capital city Addis Ababa. Based on the 2007 census conducted by the Central Statistical Agency (CSA), the population of Wolaita zone was projected to be 1,901,112. Wolaita zone is administratively divided into sixteen districts and six town administrations. Currently, there are 9 hospitals and 68 health centers. These facilities provide comprehensive emergency obstetrics and newborn care (CEmONC) and basic emergency obstetrics and newborn care (BEmONC) (Wolaita Zone Health Department Annual Report, 2019). Based on the number of pregnant/delivering mothers attending health facilities, staffing with obstetricians/midwives, and availability of diagnostic materials, hospitals were selected as the study unit, and the study was conducted from July to September 2020. The names of the hospitals with their respective total number of antenatal and delivery care service attendants in 2019 are illustrated as follows: Gesuba Primary Hospital (2113), Boditi Primary Hospital (1998), Bitena Primary Hospital (2309), Halale Primary Hospital (2458), Belle Primary Hospital (1545), WSUTRH (8242), Bombe Primary Hospital (3861), Christian Hospital (1171), and Dubo Hospital (3519). Pregnant or delivering mothers diagnosed with preeclampsia/eclampsia, gestational hypertension, and chronic hypertension with superimposed preeclampsia were included in the study as cases. Selected normotensive pregnant or delivering mother attending at each selected hospital were included in the study as control. Mothers with a history of confirmed chronic hypertension or diagnosed before 20 weeks of gestation which is greater than or equal to 140/90 mmHg and without superimposed preeclampsia and those who are critically ill and unable to communicate were excluded from the study. Mothers with a single measurement of BP greater than or equal to 140/90 mmHg and back to normal with second measurement and pending urine protein results were excluded from the study.

### 2.2. Sample Size Determination

Sample size was calculated by Epi Info 7 version 7.0.8.3 based on the following assumptions. A ratio of pregnancy-induced hypertension cases to controls 1:2, power 80, confidence level 95%, and odds ratio 4.10 by considering multiple pregnancy that had significant association with pregnancy-induced hypertension, and the proportion of exposure among controls is 4.5% from a study conducted in Ethiopia, Tigray region public hospitals [13]. We got a sample size of 257 study participants (86 cases and 171 controls). By adding a 10% nonresponse rate, finally we get a total sample size of 283 study participants (95 cases and 188 controls) (Table 1).

### 2.3. Sampling Procedure and Technique

Wolaita Sodo University Teaching and Referral Hospital, Bombe Primary Hospital, and Dubo Hospital were randomly selected. With the data gathered from Wolaita Zone Health Department, we got a total of 8,242, 3,861 and 3,519 ANC and delivery service attendants from each hospital, respectively (Wolaita Zone Health Department, 2019).

Sample size was split between these three hospitals based on proportionality of their ANC and delivery service attendants. Hence, the total annual ANC and delivery service attendants from those hospitals were 15,622. So, the sample size allocations to each hospital are illustrated as follows (Table 2).

All cases identified during antenatal and delivery services at selected hospitals were consecutively sampled until sample size fulfillment. For every case, two controls consisting of individuals from the source population who had no pregnancy-induced hypertension during attending antenatal and delivery services were selected at the selected hospitals.

### 2.4. Operational Definitions

#### 2.4.1. Cases

All mothers attending antenatal and delivery care services that had pregnancy-induced hypertension (PIH) diagnosed by gynecologists or GP or midwives at the selected hospitals

#### 2.4.2. Controls

Randomly selected mothers attending antenatal and delivery care services without pregnancy-induced hypertension (mothers who were normotensive) at the same place and time.

#### 2.4.3. Pregnancy-Induced Hypertension

A mother diagnosed as preeclampsia/eclampsia, gestational hypertension, and chronic hypertension with superimposed preeclampsia.

#### 2.4.4. Preeclampsia

A high blood pressure (SBP ≥140 mmHg and/or DBP ≥90 mmHg) plus proteinuria after 20 weeks of gestation. In the absence of proteinuria, preeclampsia is diagnosed as hypertension in association with thrombocytopenia (platelet count less than 100,000/microliter), impaired liver function (elevated blood levels of liver transaminases to twice the normal concentration), the new development of renal insufficiency (elevated serum creatinine greater than 1.1 mg/dL or a doubling of serum creatinine in the absence of other renal disease), pulmonary edema, or new-onset cerebral or visual disturbances [14, 15].

#### 2.4.5. Eclampsia

New onset of grand mal seizures in a patient with preeclampsia, without other provoking factors (such as evidence of cerebral malaria or preexisting seizure disorder) [15].

#### 2.4.6. Gestational Hypertension

Characterized by new onset of elevated blood pressure greater than or equal to SBP 140 mmHg and/or DBP 90 mmHg after 20 weeks of gestation, often near term, in the absence of accompanying proteinuria [2].

#### 2.4.7. Anemia

Hemoglobin (Hg) concentration in peripheral blood is 11 gm/dl or less [16].

#### 2.4.8. Gestational DM

A value of plasma glucose concentration is equal to or exceeds the thresholds of 92, 180, and 153 mg/dl (for fasting, one-hour, and 2-hour postglucose load glucose values, respectively) [17].

#### 2.4.9. Chronic Hypertension with Superimposed Preeclampsia

Mothers known to have hypertension before pregnancy or before 20 weeks of gestation and who had developed signs of preeclampsia after 20 weeks of gestation [15].

#### 2.4.10. Proteinuria

A urine dipstick result of +1′ and above or proteinuria ≥300 mg per 24 h. [15].

### 2.5. Data Quality Management

The questionnaire was adapted from a validated tool that was prepared in English and translated into Amharic and back to English for consistency [13]. The questions used to measure fruit and vegetable intake were adapted from Harvard University dietary assessment tool [18] A one-day intensive training was given for the data collectors about the content and administration of the questionnaire that includes the relevance of the study, purpose of the study, and confidentiality of the information informed consent and interview techniques. The data collectors had worked with close supervision of supervisors to ensure adherence for data collection procedures and supervisors checked the filled questionnaire at the end of each data collection day for completeness.

### 2.6. Data Collection Tools and Techniques

The data were collected using the pretested structured questionnaire adapted and customized from the validated questionnaire obtained from a published literature [13]. Three data collectors and one supervisor who were midwives and fluent in Amharic and Wolaitgna speaking and writing were recruited purposefully from the maternity department of each hospital. Data were collected through direct interview and supported by reviewing of medical records and reports of the study participants for the purpose of blood pressure measurement and protein in urine at the time of diagnosis. Height was measured in standing position bare foot and expressed in centimeter, while weight was recorded in kilograms with a daily calibrated weight scale and height scale. Blood pressure was measured using a calibrated sphygmomanometer and expressed in terms of mmHg.

### 2.7. Data Analysis

The collected data were manually checked for completeness and consistency, and then, data were entered to Epi Info 7 software and exported to SPSS 20 for cleaning and further analysis. Data cleaning was performed to check for accuracy, consistencies, and values. Univariate analysis using the frequency technique was performed to describe the data according to important characteristics of the study subjects. Then, the data were expressed in terms of percentages and mean. Bivariate logistic regression analysis was used to look at the crude associations between the independent variables and the dependent variable. A variable that has a *P* value of 0.25 and less was taken to multivariable logistic regression to measure the strength of associations and expressed in terms of adjusted odds ratio with 95% confidence interval by adjusting for confounders. To identify whether these variables have correlation between them, collinearity check was conducted, and variables with *P* value less than 0.05 had been declared as independent predictors of pregnancy-induced hypertension.

### 2.8. Ethical Considerations

Ethics approval was obtained from Wolaita Sodo University, College of Health Sciences, and Medicine Institutional Ethical Review Committee (IRC), and permission was obtained from the administrative office of each hospital. Verbal informed consent was taken, and confidentiality of information was ensured for each of the study participants, and participation in this research was fully voluntary.

## 3. Results

### 3.1. Sociodemographic Characteristics of Study Population

In this study, ninety-five participants with hypertensive disorder of pregnancy and 188 participants without hypertensive disorders of pregnancy were interviewed with hundred percent response rates. Of the total cases, preeclampsia/eclampsia, gestational hypertension, and chronic hypertension with superimposed preeclampsia comprised of 65 (68.4%), 16 (16.7%), and 14 (14.7%), respectively. The mean ± (SD) age of cases and controls was 26.1 ± 5.4 and 26.1 ± 4.5 years, respectively. Sixty-five percent of cases and nearly forty-eight percent of controls are rural residents. Regarding to religion and marital status, majority of study participants were Christian and married in both cases and controls. When we looked at the educational status of study participants, 15 (15.8%) cases and 52 (27.7%) of controls were illiterate. Regarding the occupation of the respondents, 41 (43.2%) cases and 107 (56.9%) of controls were housewives. When we looked at the average monthly family incomes of the respondents, 34 (35.8%) of cases and 80 (42.6%) of controls were below 1500 ETB and 38 (40%) of cases and 55 (29.3%) of controls were 3401 ETB (Table 3).

### 3.2. Obstetrics and Medical Factors

The proportion of planned pregnancies was 72 (75.8%) among cases, while it was 150 (79.8%) among controls. The proportion of multigravidas was 51 (53.7%) among cases, while it was 114 (60.6%) among controls. Proportion of cases that had the history of hypertension was found to be 45 (47.4%), while it was 20 (10.6%) in controls. Nearly 18 (20%) of cases had multiple pregnancies and 19 (10.1%) of controls had multiple pregnancies. Proportion of cases and controls that had gestational DM was 10 (10.5%) and 8 (4.3%), respectively. Appreciable number of cases had the history of abortion. Nearly 30% of cases and 13.8% of controls had the history of anemia. Above 25% of cases and a minimum number of controls had the history of kidney disease (Table 4).

### 3.3. Personal and Lifestyle Factors

Fifty-eight cases (61.1%) and twenty-six controls (13.8%) had the family history of hypertension. One-third of cases and controls had a measurement of middle upper arm circumference less or equal to 22. 1 cm. Thirty-six cases (37.9%) and twenty-six controls (13.8%) consumed alcohol during pregnancy. Less than five percent of cases and controls chewed khat (5.3% vs. 2.7%). More than eighty percent of cases and controls drunk coffee during pregnancy. More than nighty percent of cases and controls had consumed coffee daily, and three-fourths of cases and controls drunk less or equal to two cups of coffee daily. More than eighty percent of cases and nighty percent of controls had taken fruit and vegetables in their diet (Table 5).

### 3.4. Risk Factors of Hypertensive Disorders of Pregnancy

Bivariate analysis was run in the unconditional logistic regression to check the association between dependent and independent variables. Accordingly, rural residence, illiterate, history of previous PIH, multiple pregnancy, gestational DM, history of abortion, pregestational DM, history of anemia, history of kidney disease, family history of HTN, and alcohol consumption were found to be a potential risk factors for hypertensive disorders of pregnancy. In addition, the bivariate analysis revealed that eating vegetable and fruit was found to be a potential protective factor for hypertensive disorders of pregnancy.

Variables which were associated with the outcome variable in the bivariate analysis (*P* ≤ 0.25) were taken to the multivariable analysis. After adjusting for possible confounding factors in the unmatched unconditional logistic regression, only rural residence, illiterate, history of previous PIH, history of kidney disease, family history of hypertension, and vegetable intake were independent predictors of pregnancy-induced hypertension. Mothers who live in the rural area were at a greater odd of developing PIH as compared to mothers residing in urban areas (AOR: 2.25, 95% CI: 1.09–4.65). Illiterate mothers were also at greater risk of developing PIH as compared to literate women (AOR: 3.12, 95% CI: 1.20–8.08). The risk of developing pregnancy-induced hypertension among mothers who had the history of PIH were 6.62 times higher than mothers who had no history of PIH (AOR: 6.62, 95% CI: 2.48–17.71). Mothers who had the history of kidney disease were also at a greater odd of developing PIH as compared to mothers who had no history of kidney disease (AOR: 3.14, 95% CI: 1.05–9.38). The family history of hypertension is also an independent predictor of PIH. The risk of developing PIH among mothers who had the family history of hypertension were 5.59 times higher than mothers who had no family history of hypertension (AOR: 5.59, 95% CI: 2.73–11.45). Vegetable intake was the only independent protective factor of pregnancy-induced hypertension. There is 77% reduced odds of developing PIH if mothers had taken vegetable in their diet (Table 6).

## 4. Discussion

Mothers who live in the rural area were at a greater odd of developing pregnancy-induced hypertension as compared to mothers who live in urban areas (AOR: 2.25, 95% CI: 1.09–4.65). This is consistent with the findings of earlier studies conducted in Jimma [19] and Tigray regions [13] of Ethiopia. A study conducted in Jimma town identified that mothers who live in the rural area were 5.3 times at more risk of developing pregnancy-induced hypertension than urban residents (AOR: 5.3, 95% CI: 1.5–18.5). In the Tigray region, also rural residents were 3.7 times at more risk of developing pregnancy-induced hypertension as compared to urban residents (AOR: 3.7, 95% CI: 1.9–7.1). This might justify those women at rural area accessing less healthcare infrastructures such as health facilities, roads, transportation, and media for health information and communication for seeking maternal health services including preconception care as compared to urban residents. But this study result was inconsistent with a cross-sectional study conducted in Ghana [20] that showed urban residents were at a greater risk of developing pregnancy-induced hypertension than rural residents. This might be due to the difference in the study area, health policies of the country, lifestyle of urban women of Ghana, and health seeking behavior of Ghana's rural community.

Similarly, this study showed that women who were illiterate were positively associated with the development of pregnancy-induced hypertension (AOR: 3.12, 95% CI: 1.20–8.08). This result is consistent with a study conducted in eastern Sudan [21] and Jordan [22] that showed women with a lower educational level and/or illiterate were at higher risk of developing pregnancy-induced hypertension. A previous case-control study conducted in Kombolcha, Ethiopia [23], also revealed that mothers who did not read and write were at a greater odd of developing pregnancy-induced hypertension as compared to mothers who can read and write. In agreement with this, a systematic and meta-analysis conducted in sub-Saharan African countries revealed that women who had lower educational level put women at a higher risk of developing pregnancy-induced hypertension [24].

Women in SSA including Ethiopia are mostly uneducated and may not understand how important early identification of ANCs visit is; therefore, they are likely to develop the disorder and negative health outcomes [24].

This study also showed that the history of previous pregnancy-induced hypertension is an independent risk factor for the development of pregnancy-induced hypertension (AOR: 6.62, 95% CI: 2.48–17.71). Mothers who had the history of pregnancy-induced hypertension had 6.62 times more risk of developing pregnancy-induced hypertension as compared to mothers who had no history of pregnancy-induced hypertension.

Previous studies conducted in Cameroon [25], India [26], Jordan [22], and Nigeria [27] reported that there was a higher risk of developing PIH among women who had the history of pregnancy-induced hypertension that were congruent to this study result.

This study result was also consistent with previous studies conducted in Jijiga [28], Kombolcha [23], and Addis Ababa [29], Ethiopia. The level of risk of developing pregnancy-induced hypertension is somewhat similar to the study conducted in Kombolcha (AOR, 4.22, 95% CI: 2.06–8.64) and Addis Ababa (OR: 4.28, 95% CI: 1.61–11.43), but it is much lower than the study conducted in Jijiga town (AOR = 19.3, 95% CI:  5.2–72.1) as compared to this study result. This discrepancy might be due to the methodological difference used by the researcher. This study result implies women who had pregnancy-induced hypertension on their prior pregnancy need to have a focus and could be an acceptable means of screening for pregnancy-induced hypertension.

A multivariable analysis showed that the history of kidney disease was an independent risk factor for the development of pregnancy-induced hypertension (AOR: 3.14, 95% CI: 1.05–9.38). Mothers who had the history of kidney disease were 3.16 times at more risk of developing pregnancy-induced hypertension as compared to their counterparts. A cross-sectional study conducted in Jimma town also showed that women who had a history of kidney disease were 3.97 (95% CI: 1.36–11.56) times at more risk of developing pregnancy-induced hypertension than women who had no history of kidney disease [30]. Similar study conducted in southwest Ethiopia also revealed that mothers who had the history of kidney disease had 3.32 (95% CI: 1.04–10.58) times at more risk of developing pregnancy-induced hypertension as compared to mothers who had no previous history of kidney diseases [19]. This may be due to that renal disease interferes with salt excretion leading to volume overload and subsequent hypertension [2, 3].

The present study also reported a statistically significant association between the family history of hypertension and pregnancy-induced hypertension (AOR: 5.59, 95% CI: 2.73–11.45). Mothers who had the family history of hypertension were 5.59 times at more risk of developing pregnancy-induced hypertension than mothers who do not have the family history of hypertension. Prior cross-sectional studies conducted in Jimma, Jijiga, southwest, Ethiopia, and Gondar strongly supported this finding [19, 23, 28, 30, 31]. A previous study conducted in southern Brazil [14] reported that mothers with the family history of hypertension was 3.88 (95% CI: 1.77–8.46) times at more higher risk of developing pregnancy-induced hypertension as compared to their counterparts. This might be as a result of genetic inheritance which causes the dilator and constrictor imbalances at microvasculature levels [2, 3].

Multiple pregnancies were associated with the development of pregnancy-induced hypertension in the bivariate analysis, but its effect was vanished in the multivariate analysis that contradicts with previous reports. These reports showed that multiple pregnancies were an independent risk factor for the development of pregnancy-induced hypertension [13, 21, 22, 27, 32]. This might be due to exposure of excessively abundant trophoblastic tissue and possibly from psychological and physiological stress that develops in the women because of the multiple pregnancies [3].

The most peculiar parts of this study identify that vegetable intake dramatically reduces the odds of developing pregnancy-induced hypertension (AOR: 0.23, 95% CI: 0.06–0.79). Seventy-seven percent of odds of developing pregnancy-induced hypertension can be prevented by eating adequate vegetables. This might be due to the fact that fruit and vegetable dietary which are rich of vitamin C and vitamin E are associated with pregnancy-induced hypertension reduction. The prevention of these vitamins from PIH development is due their antioxidant effects [2, 33].

A study conducted on dietary factors associated with preeclampsia or eclampsia among women in delivery care services in Addis Ababa, Ethiopia, revealed that 5% (AOR: 0.95, 95% CI: 0.01–0.7) of odds of developing pregnancy-induced hypertension can be prevented by varying vegetables in women daily diet [33]. In addition to this, a case-control study conducted in Tigray region showed that women who ate less vegetable had a greater odd of developing pregnancy-induced hypertension [13]. Likewise, in the bivariate analysis, adequate fruit intake was identified as a protective factor (COR: 0.26, 95% CI: 0.10–0.70) that is similar with previous studies conducted in Addis Ababa [33] and Tigray region [13]. Fruit and vegetable intake during pregnancy could be taken as a protective factor for pregnancy-induced hypertension in this study.

### 4.1. Strengths and Limitations of the Study

The strength of this study was its representativeness, such that it covers zonal population, and it is also the first study in Wolaita zone that could be a reference to other researchers. Eventhough it employed the stronger study design, the findings of this study should be viewed in light of the following limitations. Any random and systematic measurement error in self-reported data might attenuate the associations observed in this study. Assessment of risk factors was made at diagnosis; hence, recall bias is inevitable.

## 5. Conclusion

This study identified multiple predictors of pregnancy-induced hypertension. These predictors include rural residence, illiterate, history of pregnancy-induced hypertension, history of kidney disease, family history of hypertension, and adequate vegetable intake. Early recognition and management of these factors at the community level and in preconception care settings could reduce significant amount of maternal morbidity and mortality.

## Figures and Tables

**Table 1:** Factor analysis to determine sample size using different associated factors of pregnancy-induced hypertension.

Associated factors	Power	Cases to controls ratio	Odds ratio	Percent of controls exposed (%)	95% CI	Sample size			Reference
						Cases	Controls	Total	
Residence	80	1 : 2	3.7	34.5	(1.9–7.1)	33	66	99	[13]
Fruit use	80	1 : 2	5.1	12.3	(2.4–11.1)	30	59	89	
Multiple pregnancy	80	1 : 2	4.1	4.5	(1.8–9.6)	86	171	257	
BMI of mothers ≥25 Kg/m^2^ (prepregnancy)	80	1 : 2	5.5	2.7	(1.1–27.6)	84	167	251	

**Table 2:** Sample size allocations for the selected hospitals, Wolaita zone, 2019 (Wolaita Zone Health Department Annual Report, 2019).

Hospitals	Number of annual maternal service attendants (ANC and delivery)	Sample size			Remarks
		Cases	Controls	Total	
WSUTRH	8,242	50	99	149	
Bombe Primary Hospital	3,861	24	46	70	
Dubo Hospital	3,519	21	43	64	

**Table 3:** Sociodemographic characteristics of mothers with and without hypertensive disorders of pregnancy-induced hypertension in Wolaita zone, southern Ethiopia, 2020.

Variables		PIH		Crude OR (95% CI)	*P* value
		Cases	Controls		
		Number (%)	Number (%)		
Age	≤20 years	19 (20.0)	23 (21.2)	1.86 (0.95–3.64)	0.70
	21–34 years	67 (70.5)	151 (80.3)	1	
	≥35 years	9 (9.5)	14 (7.4)	1.44 (0.59–3.51)	0.412

Residence	Urban	34 (35.8)	98 (52.1)	1	
	Rural	61 (64.2)	90 (47.9)	1.95 (1.17–3.24)	0.010

Marital status	Married	89 (93.7)	181 (96.3)	1	
	Unmarried	6 (6.3)	7 (3.7)	1.74 (0.56–5.34)	0.331

Religion	Christian	92 (96.8)	181 (96.3)	1	
	Muslim	3 (3.2)	7 (3.7)	1.18 (0.30–4.69)	

Educational status	Did not read and write	15 (15.8)	52 (27.7)	2.03 (1.07–3.85)	0.028
	Literate	80 (84.2)	136 (72.3)	1	

Occupation	Housewives	41 (43.2)	107 (56.9)	1.74 (0.86–2.86)	0.129
	Others	54 (56.8)	81 (43.1)	1	

Spousal education status	Illiterate	11 (11.6)	22 (11.7)	1.01 (0.46–2.18)	0.976
	Literate	84 (88.4)	166 (88.3)	1	

Family size	01–02	45 (47.4)	77 (41.0)	1	
	03–04	35 (36.8)	80 (42.6)	0.74 (0.43–1.28)	0.294
	≥05	15 (15.8)	31 (16.5)	0.82 (0.40–1.69)	0.606

Average family income	≤1500	34 (35.8)	80 (42.6)	1	
	1500–3400	23 (24.2)	53 (28.2)	1.02 (0.54–1.92)	0.948
	≥3401	38 (40)	55 (29.3)	1.62 (0.91–2.89)	0.098

**Table 4:** Obstetrics and medical characteristics of mothers with/without hypertensive disorders of pregnancy in Wolaita zone, southern Ethiopia, 2020.

Variables		PIH		COR (95% CI)	*P* value
		Case	Control		
		Number (%)	Number (%)		
Pregnancy status	Planned	72 (75.8)	150 (79.8)	1	
	Unplanned	23 (24.2)	38 (20.2)	1.26 (0.70–2.27)	0.440

Gravidity	Primigravida	44 (46.3)	74 (39.4)	1	
	Multigravida	51 (53.7)	114 (60.6)	0.75 (0.45–1.23)	0.263

History of previous PIH	Yes	45 (47.4)	20 (10.6)	7.56 (4.09–13.97)	<0.0001
	No	50 (52.6)	160 (89.4)	1	

Multiple pregnancy	Yes	18 (18.9)	19 (10.1)	2.07 (1.03–4.18)	0.040
	No	77 (81.1)	169 (89.9)	1	

Gestational DM	Yes	10 (10.5)	8 (4.3)	2.64 (1.00–6.94)	0.048
	No	85 (89.5)	180 (95.7)	1	

Average pregnancy interval (*n* = 162)	<18 months	4 (4.2)	7 (3.7)	1.81 (0.47–6.96)	0.385
	18–24 months	17 (17.9)	54 (28.7)	1	
	≥25 months	26 (27.4)	54 (28.7)	1.52 (0.74–3.13)	0.246

Hx of abortion	Yes	38 (40.0)	42 (22.3)	2.31 (1.35–3.95)	0.002
	No	57 (60.0)	146 (77.7)	1	

Pregestational DM	Yes	16 (16.8)	8 (4.3)	4.55 (1.87–11.80)	0.001
	No	79 (83.2)	180 (95.7)	1	

Hx of anemia in current pregnancy	Yes	29 (30.5)	26 (13.8)	2.73 (1.50–4.99)	0.001
	No	66 (69.5)	162 (86.2)	1	

Hx of kidney disease	Yes	24 (25.3)	9 (4.8)	6.72 (2.97–15.17)	<0.0001
	No	71 (74.7)	179 (95.2)	1	

Hx of asthma	Yes	3 (3.2)	6 (3.2)	0.98 (0.24–4.04)	0.988
	No	92 (96.8)	182 (96.8)	1	

Hx of rheumatic arthritis	Yes	5 (5.3)	7 (3.7)	1.43 (0.44–4.65)	0.546
	No	90 (94.7)	181 (96.3)	1	

**Table 5:** Dietary, familial, and lifestyle characteristics of mothers with/without hypertensive disorders of pregnancy in Wolaita zone, southern Ethiopia, 2020.

Variables		PIH		COR (95% CI)	*P* value
		Cases	Controls		
		Number (%)	Number (%)		
Family Hx of HTN	Yes	58 (61.1)	26 (13.8)	9.76 (5.44–17.52)	<0.0001
	No	37 (38.9)	162 (86.2)	1	

MUAC	≤22.1 cm	30 (31.6)	65 (34.6)	1	
	>22.1 cm	65 (68.4)	123 (65.4)	1.14 (0.67–1.93)	0.614

Alcohol consumption	Yes	36 (37.9)	26 (13.8)	3.80 (2.11–6.83)	<0.0001
	No	59 (62.1)	162 (86.2)	1	

Chewing chat	Yes	5 (5.3)	5 (2.7)	2.03 (0.57–7.20)	0.272
	No	90 (94.7)	183 (97.3)	1	

Drinking coffee	Yes	84 (88.4)	149 (79.3)	1.99 (0.97–4.10)	0.06
	No	11 (11.6)	39 (20.7)	1	

Frequency of drinking coffee (*n* = 233)	Daily	77 (91.7)	145 (97.3)	3.29 (0.93–11.60)	0.063
	Weekly	7 (8.3)	4 (2.7)	1	

Amount of coffee (*n* = 233)	≤2 cups	71 (84.5)	117 (78.5)	1	
	3 or more cups	13 (15.5)	32 (21.5)	0.66 (0.33–1.360)	0.267

Fruit intake	Yes	83 (87.4)	181 (96.3)	0.26 (0.10–0.70)	0.008
	No	12 (12.6)	7 (3.7)	1	

Vegetable intake	Yes	80 (84.2)	182 (96.8)	0.17 (0.06–0.47)	0.001
	No	15 (15.8)	6 (3.2)	1	

**Table 6:** Multivariable unconditional logistic regression analysis of predictors of hypertensive disorders of pregnancy in Wolaita zone, southern Ethiopia, 2020.

Variables		PIH		Odds ratio (95% CI)	
		Cases	Controls		
		No (%)	No (%)	Crude OR	AOR
Residence	Urban	34 (35.8)	98 (52.1)	1	
	Rural	61 (64.2)	90 (47.9)	1.95 (1.17–3.24)	2.25 (1.09–4.65)^*∗*^

Educational status	Did not read and write	15 (15.8)	52 (27.7)	2.03 (1.07–3.85)	3.12 (1.20–8.08)^*∗*^
	Literate	80 (84.2)	136 (72.3)	1	

Occupation	Housewives	41 (43.2)	107 (56.9)	1.57 (1.34–4.32)	0.34 (0.01–2.64)
	Others	54 (56.8)	81 (43.1)	1	

Hx of previous PIH	Yes	45 (47.4)	20 (10.6)	7.56 (4.09–13.97)	6.62 (2.48–17.71)^*∗∗*^
	No	50 (52.6)	160 (89.4)	1	

Multiple pregnancy	Yes	18 (18.9)	19 (10.1)	2.07 (1.03–4.18)	0.79 (0.25–2.46)
	No	77 (81.1)	169 (89.9)	1	

Gestational DM	Yes	10 (10.5)	8 (4.3)	2.64 (1.00–6.94)	0.78 (0.18–3.38)
	No	85 (89.5)	180 (95.7)	1	

Hx of abortion	Yes	38 (40.0)	42 (22.3)	2.31 (1.35–3.95)	0.79 (0.33–1.83)
	No	57 (60.0)	146 (77.7)	1	

Pregestational DM	Yes	16 (16.8)	8 (4.3)	4.55 (1.87–11.80)	1.10 (0.30–3.95)
	No	79 (83.2)	180 (95.7)	1	

Hx of anemia	Yes	29 (30.5)	26 (13.8)	2.73 (1.50–4.99)	1.07 (0.45–2.53)
	No	66 (69.5)	162 (86.2)	1	

Hx of kidney disease	Yes	24 (25.3)	9 (4.8)	6.72 (2.97–15.17)	3.14 (1.05–9.38)^*∗*^
	No	71 (74.7)	179 (95.2)	1	

Family Hx of HTN	Yes	58 (61.1)	26 (13.8)	9.76 (5.44–17.52)	5.59 (2.73–11.45)^*∗∗*^
	No	37 (38.9)	162 (86.2)	1	

Alcohol consumption	Yes	36 (37.9)	26 (13.8)	3.80 (2.11–6.83)	2.08 (0.95–4.54)
	No	59 (62.1)	162 (86.2)	1	

Fruit intake	Yes	83 (87.4)	181 (96.3)	0.26 (0.10–0.70)	0.60 (0.15–2.31)
	No	12 (12.6)	7 (3.7)	1	

Vegetable intake	Yes	80 (84.2)	182 (96.8)	0.17 (0.06–0.47)	0.23 (0.06–0.79)^*∗*^
	No	15 (15.8)	6 (3.2)	1	

## Data Availability

The data generated or analyzed during this study are included within the article.
